# Real-world survey on utilization of central antitussives and its health impact in patients with subacute and chronic cough in Japan

**DOI:** 10.1038/s41598-025-30832-6

**Published:** 2025-12-08

**Authors:** Junpei Saito, Shotaro Maeda

**Affiliations:** 1 Department of Pulmonary Medicine, Fukushima Medical University, Fukushima, Japan; 2 Medical Affairs, Kyorin Pharmaceutical Co., Ltd., Tokyo, Japan

**Keywords:** Adverse event, Central antitussives, Chronic cough, Comorbidity, Real world data, Subacute cough, Medical research, Risk factors

## Abstract

Despite guideline recommendations against the long-term use of central antitussives, actual clinical practice remains unclear. We aimed to investigate prescription patterns of central antitussives in patients with cough using Japanese claims data. In this retrospective cohort study, we evaluated patients aged ≥ 20 years with subacute and chronic cough from IQVIA Claims database. The primary outcome was the number of days central antitussives were prescribed. Additionally, we examined co-occurrence patterns of comorbidities and adverse events, specifically nausea and constipation, in relation to the duration of central antitussive prescription. Among 10,385 patients with subacute/chronic cough, 4431 (42.7%) were prescribed central antitussives. A longer duration of central antitussives prescription was significantly associated with a higher frequency of comorbidities. Specifically, the proportion of bronchial asthma/cough variant asthma, allergic rhinitis/chronic rhinosinusitis, and gastroesophageal reflux disease increased with the number of prescribed days of central antitussive. Adjusted odds ratio (95%CI) for constipation and nausea were 1.80 (1.18–2.76) and 2.06 (1.15–3.70), respectively, in patients prescribed central antitussives for > 57 days compared to those prescribed for 0–28 days. Appropriate treatment of comorbidities and prescription of central antitussives are warranted.

**Supplementary Information:**

The online version contains supplementary material available at 10.1038/s41598-025-30832-6.

## Introduction

Cough is one of the most frequent complaints in daily medical practice. Subacute cough (lasting for 3–8 weeks) and chronic cough (persisting for more than 8 weeks) can frequently cause significant social burdens, including impairments in quality of life, work performance, and sleep disturbances^1,2^. A systematic review investigating the prevalence of chronic cough in adults reported prevalence rates of 11.0%, 12.7%, and 4.4% in the United States, Europe, and Asia, respectively^3^. The major causes of chronic cough include bronchial asthma (BA), cough-variant asthma (CVA), upper airway cough syndrome (UACS), and gastroesophageal reflux disease (GERD)^4–8^. There are few reports on the prevalence of subacute cough, and although it varies from 4.7% to 48.4% depending on the background and region, the most common cause is thought to be post-infection cough^9–11^. In patients with subacute cough, it is recommended to first determine if it is post-infectious and, if non-infectious, to manage it similarly to chronic cough ^1^. Therefore, the concept of an anatomic diagnostic protocol that assumes causative diseases and specific treatment based on the anatomic localisation of cough receptors and afferent sensory nerves is recommended in cough guidelines in both Western countries and Japan^1,12,13^.

Therapeutic agents for cough treatment are classified as those that act centrally or peripherally. Most specific treatments based on anatomical diagnostic protocols are classified as peripherally acting agents. Conversely, centrally acting antitussives, such as codeine phosphate and dextromethorphan, suppress cough at the central level, regardless of causes^14^. Thus, central antitussives may suppress “necessary cough” as a physiological defense reaction and are associated with various side effects such as constipation, nausea, and drowsiness. Furthermore, there is a risk of fatal outcomes in patients with respiratory depression^15–17^. Therefore, current guidelines do not recommend routine use of central antitussives for all cough; rather, they should be considered for severe, burdensome cough after evaluation and targeted treatment of underlying causes, with short-term use and monitoring. When appropriately selected and monitored, the use of central antitussives can provide clinically meaningful relief, and adverse effects are generally manageable in practice^13^. However, reports on the actual proportions of antitussives prescribed by medical institutions are limited^18^. In Japan, only one report has investigated prescription patterns among patients with chronic cough using claims data^19^. However, because this study did not evaluate the number of days prescribed or the proportion of days prescribed, it remains unclear how adequately central antitussives are prescribed in the real world.

Suppose central antitussives are inappropriately prescribed to patients with subacute and chronic cough and there is concern about adverse events caused by long-term prescription. In that case, this investigation may contribute to a more appropriate diagnosis and treatment of cough based on anatomical diagnostic procedures, which may ultimately lead to precision medicine^20^. Therefore, this study aimed to investigate the real-world prescription patterns, duration and adverse events of central antitussive use among patients with subacute and chronic cough by retrospectively analyzing a large-scale Japanese claims database.

## Results

### Patient characteristics

Patient characteristics were described by the number of prescribed days of central antitussives during the follow-up period (Table 1). Female and male patients accounted for 61.4% and 38.6%, respectively, and general internal medicine accounted for 61.5% of the total number of patients. Approximately half (60.0%) of the patients had BA/CVA, followed by allergic rhinitis (AR)/chronic sinusitis (CRS) (51.8%) and GERD (26.8%). Descriptive statistics for other comorbidities and medications are presented in Supplementary Tables S1 and S2.

**Table 1:** Baseline characteristics.

Variables	Prescribed days of central antitussive*							
	0–28 days (n = 9469)		29–56 days (n = 524)		≥ 57 days (n = 392)		Total (N = 10,385)	
Sex, n (%)								
Female	5830	61.6%	317	60.5%	231	58.9%	6378	61.4%
Male	3639	38.4%	207	39.5%	161	41.1%	4007	38.6%
Age								
Mean (SD)	44.6	12.2	43.3	10.9	46.6	12.6	44.6	12.2
Department, n (%)								
General Internal Medicine	5827	61.5%	325	62.0%	238	60.7%	6390	61.5%
Otolaryngology	1103	11.6%	59	11.3%	32	8.2%	1194	11.5%
Respiratory	586	6.2%	55	10.5%	48	12.2%	689	6.6%
Department size (beds), n (%)								
0–19	7553	79.8%	427	81.5%	282	71.9%	8262	79.6%
≥ 20	1741	18.4%	91	17.4%	104	26.6%	1936	18.6%
Unknown	175	1.8%	6	1.1%	6	1.5%	187	1.8%
Comorbidities, n (%)								
BA/CVA	5497	58.1%	405	77.3%	328	83.7%	6230	60.0%
AR/CRS	4809	50.8%	296	56.5%	271	69.1%	5376	51.8%
GERD	2452	25.9%	166	31.7%	165	42.1%	2783	26.8%
Medications, n (%)								
ICS	673	7.1%	47	9.0%	47	12.0%	767	7.4%
ICS/LABA	4083	43.1%	339	64.7%	275	70.2%	4697	45.2%
ICS/LABA/LAMA	401	4.2%	60	11.5%	56	14.3%	517	5.0%
H1RA	6294	66.5%	437	83.4%	316	80.6%	7047	67.9%
LTRA	4417	46.6%	362	69.1%	276	70.4%	5055	48.7%
PPI	2357	24.9%	167	31.9%	160	40.8%	2684	25.8%
Prokinetics	1924	20.3%	151	28.8%	104	26.5%	2179	21.0%
Prescription of central antitussive (follow-up period), days								
Mean (SD)	4.3	7	39.8	8.3	136.0	87.7	11.1	31.8
Median (IQR)	0	0–7	38	33–45	100	72–167	0	0–10

* Includes duplicates within subgroups.

AR, allergic rhinitis; BA, bronchial asthma; CRS, chronic rhinosinusitis; CVA, cough variant asthma; GERD, gastroesophageal reflux disease; H1RA, histamine H1-receptor antagonist; ICS, inhaled corticosteroid; IQR, interquartile range; LABA, long-acting β2-agonist; LAMA, long-acting muscarinic antagonist; LTRA, leukotriene receptor antagonist; PPI, proton pump inhibitor; RCC, refractory chronic cough; SD, standard deviation.

### Prescription of central antitussive

**Fig. 1:**
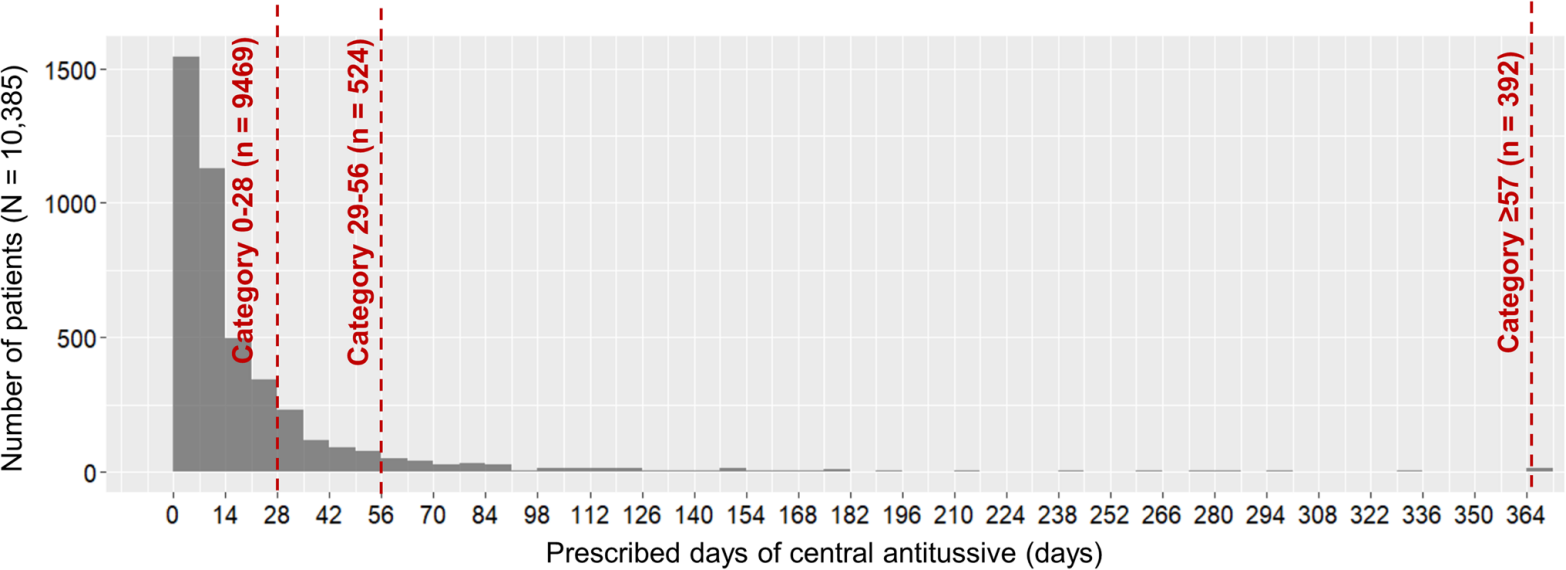
Distribution of the list of prescription days for central antitussives. The horizontal axis of the figure shows the cumulative number of prescribed days of central antitussives during the follow-up period divided into 7-day intervals. The bars indicate the number of patients, and the dotted line indicates the cutoff for the cumulative number of days prescribed (Table 3). We omitted bars for patients who did not have a prescription for central antitussives.

Of the 10,385 patients included in the analysis, 4431 (42.7%) were prescribed central antitussives, while the remaining 5954 (57.3%) did not receive central antitussives. Among those who prescribed central antitussives, 3515 patients (33.8%) were prescribed central antitussives for 1–28 days, 524 (5.0%) for 29–56 days, and 392 (3.8%) for ≥ 57 days. Thereafter, as for the purposes of analysis, patients who were not prescribed central antitussives were grouped with those prescribed for 0–28 days (9469 patients; 91.2%), because the risk of adverse effects would not substantially differ between those with no prescription and those treated for only a short duration. The distribution of central antitussives prescribed every 7 days is shown in Fig. 1.

### Co-occurrence patterns of comorbidity and medications

**Fig. 2:**
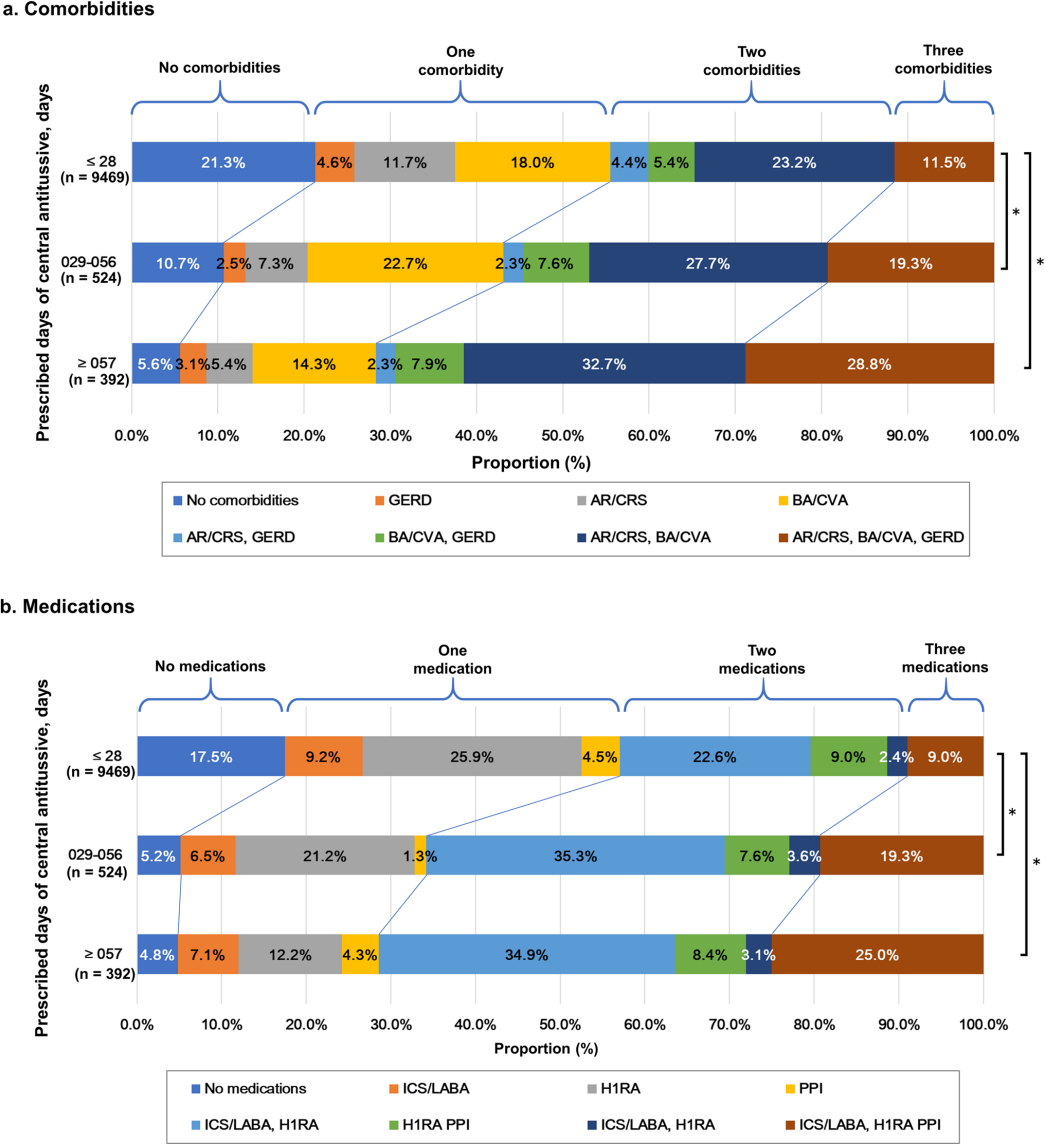
Co-occurrence patterns of comorbidity and medications. * *P* < .001. *P*-values were derived using an ordinal logistic regression model. a. Comorbidity, b. Medication. AR, allergic rhinitis; BA, bronchial asthma; CRS, chronic rhinosinusitis; CVA, cough variant asthma; GERD, gastroesophageal reflux disease; H1RA, histamine H1-receptor antagonist; ICS, inhaled corticosteroid; IQR, interquartile range; LABA, long-acting β2-agonist; LAMA, long-acting muscarinic antagonist; LTRA, leukotriene receptor antagonist; PPI, proton pump inhibitor.

The proportion of patients with comorbid BA, GERD, and AR divided by the number of days of prescription of central antitussives was 11.5%, 19.3%, and 28.8% for 0–28, 29–56, and ≥ 57 days, respectively. The ordinal logistic regression model analyzing the relationship between the number of prescription days and the number of comorbidities showed that the groups with 29–56 and ≥ 57 days prescriptions had estimates (SE) of 0.58 (0.08) and 1.17 (0.09) for the 0–28 days group, with the larger number of prescription days group containing a significantly larger number of complication categories (*P* < 0.001 and *P* < 0.001, respectively; Fig. 2a). The proportion of patients who received longer prescriptions for central antitussives increased with an increase in the number of comorbidities, especially when BA/CVA, AR/CRS, and GERD were combined. In contrast, the proportion of patients with GERD alone, GERD and BA/CVA, or GERD and AR/CRS did not differ substantially in terms of the number of days of central antitussive prescription. There were no marked differences in the vocal cord dysfunction (VCD) or obstructive sleep apnoea (OSA) between the groups (Supplementary Table S1). Similar results were observed in the pattern of co-occurrence of medications, and the ordinal logistic regression model analyzing the relationship between the number of prescription days and the number of medications showed that the estimates (SE) for the groups with 29–56 and ≥ 57 days of prescriptions were 0.95 (0.08) and 1.22 (0.10) for the group with 0–28 days, and the group with a larger number of prescription days significantly included a higher level of co-occurrence of medications (*P* < 0.001 and *P* < 0.001, respectively; Fig. 2b).

### Association with adverse events

In the analysis of all central antitussives and adverse events, patients given a prescription for ≥ 57 days had significantly higher odds of constipation than those given the prescription for 0–28 days (odds ratio [OR], 1.80; 95% confidence interval [95%CI], 1.18–2.76), and significantly higher odds of nausea in the ≥ 57 days groups (OR, 2.06; 95%CI, 1.15–3.70; Table 2). The results of the subgroup analysis, categorized by opioid and non-opioid central antitussives, showed that for opioid central antitussives, the ORs of constipation were significantly higher in the ≥ 57 days group (OR 2.46; 95%CI 1.29–4.71); for the non-opioid antitussives, the odds of nausea was higher in the ≥ 57 days group (OR 2.49; 95%CI 1.24–5.01; Table 2). Sensitivity analysis also showed similar results, with an increase in adverse events of constipation in the ≥ 57 days group for opioid central antitussives (OR 2.70; 95% CI 1.41–5.18), while non-opioid central antitussives showed an increased odds ratio for nausea in the ≥ 57 day group (OR 2.78; 95% CI 1.37–5.66) (Supplementary Table S6.)

**Table 2:** Associations between prescribed days of central antitussives and adverse events from multivariable Logistic regression analyses.

Event type	Prescriptions (days)	Reference (days)	OR	LCL	UCL	*P* values
All central antitussives						
Model 1: Constipation	29–56	0–28	1.38	0.92	2.08	0.124
	≥ 57	0–28	1.80	1.18	2.76	0.006
Model 2: Nausea	29–56	0–28	1.44	0.80	2.62	0.226
	≥ 57	0–28	2.06	1.15	3.70	0.016
Opioid central antitussives						
Model 1: Constipation	29–56	0–28	1.50	0.81	2.77	0.201
	≥ 57	0–28	2.46	1.29	4.71	0.006
Model 2: Nausea	29–56	0–28	2.02	0.85	4.79	0.113
	≥ 57	0–28	1.47	0.58	3.71	0.414
Non-opioid central antitussives						
Model 1: Constipation	29–56	0–28	1.01	0.59	1.73	0.976
	≥ 57	0–28	1.46	0.81	2.62	0.211
Model 2: Nausea	29–56	0–28	1.22	0.52	2.87	0.641
	≥ 57	0–28	2.49	1.24	5.01	0.011

P values were derived from multivariable logistic regression model, adjusted by age, sex, frequency of diagnosis of cough, comorbidities (BA/CVA, AR/CRS, GERD). In addition, model was adjusted by frequency of diagnosis of nausea in look-back period for model-1, constipation for model-2, respectively.

LCL, 95% confidence lower limit; OR, odds ratio; UCL, 95% confidence upper limit.

## Discussion

This study retrospectively investigated the use of central antitussives and their health effects, using claims data. The overall proportion of patients with subacute or chronic cough among those diagnosed with cough at least once during the period under review was 5.92% (10,385 out of 175,239). Of these, 33.8% received central antitussives for ≤ 4 weeks, while 8.8% received central antitussives for over a month. The higher the complication rate, the higher the prescription rate of central antitussives. Furthermore, the longer the treatment duration with central antitussives, the higher the frequency of common adverse events, such as constipation and nausea.

In the present study, the proportion of patients with subacute or chronic cough was 5.92%. The prevalence of subacute and chronic cough in epidemiological surveys varies depending on the country surveyed, target population, and quality of the healthcare system. There are very few reports on the prevalence of subacute cough, whereas the worldwide prevalence of chronic cough was approximately 9.6% in a comprehensive systematic review and meta-analysis^3^. The prevalence of chronic cough in Asia is 4.4%, which is relatively lower than that in other regions, such as Europe and Oceania. According to an Internet survey in Japan conducted by Tobe et al., the prevalence of chronic cough was 4.29%^21^. The findings of these studies on chronic cough are similar to ours, indicating that the participants in this study reflected the actual situation of patients with chronic coughs in Japan.

The proportion of central antitussives prescribed to the entire population was 42.7%, which was higher than anticipated. Community-based surveys have found the proportion of central antitussives prescribed to be 11.9–28.8%^18,22–24^. In contrast, the rate of prescribing central antitussives in specialist clinics (54.0–58.9%) was higher than that in primary care clinics^22,25^. A possible reason for the higher prescription rate of central antitussives in this study could be the implementation of a national health insurance system in Japan, resulting in easy access to specialists and primary care clinics.

In terms of the duration of prescribing central antitussives, 33.8% of the patients were prescribed central antitussives for ≤ 4 weeks, while 392 out of 10,385 patients (3.8%) received central antitussives for > 8 weeks, and the mean (standard deviation [SD]) duration was 136.0 ± 87.7 days. Notably, 4.8% of the patients had been prescribed only central antitussives for ≥ 57 days without treatment for the underlying specific conditions, assuming that they may not have been managed appropriately according to the anatomical diagnostic procedure, including lifestyle modifications. Alternatively, these patients may have had an unexplained chronic cough (UCC) where the cause of cough remains unidentified despite appropriate diagnostic and therapeutic approach^26^. To the best of our knowledge, only one study has quantitatively estimated the number of days of central antitussive prescriptions in medical institutions^27^. Among the 1,233 patients with chronic cough, 173 (14%) received codeine for > 8 weeks. The mean (SD) number of days for which codeine was prescribed was 51.4 (66.8), and the upper fifth percentile was 200 days. Furthermore, none of the patients were prescribed only central antitussives. This indicates that a certain number of patients may be prescribed central antitussives for symptomatic relief rather than for further exploration of the cause of cough based on the anatomic diagnostic protocol. As recommended by the guidelines^13^, it is important to educate physicians and patients that the administration of central antitussives should be short-term and that efforts should be made to treat the underlying conditions as much as possible.

In this study, the group with more central antitussive prescription days had a higher proportion of BA/CVA, AR/CRS, and GERD complications (Table 1) and a higher proportion of co-occurrences (Fig. 2a). This finding may reflect several clinical scenarios, including suboptimal control of underlying diseases, poor adherence to treatment, inadequate diagnostic evaluation, enhanced cough reflex sensitivity, or a combination of these factors. However, given the limitations of our retrospective design, definitive conclusions cannot be drawn. Future prospective investigations based on medical records and laboratory testing data in routine clinical practice rather than claim database are required to clarify this finding. Notably, some patients may continue to suffer from persistent cough despite being adequately treated for underlying conditions as well as cough-related comorbidities. These individuals may represent cases of refractory chronic cough, where symptoms persist despite appropriate diagnosis and treatment. Recently, it has been suggested that such patients may have coexisting cough hypersensitivity syndrome (CHS), which induces cough with low levels of irritation due to increased sensitivity^28^. The cause of CHS is considered to be hypersensitivity of the afferent sensory nerves and dysfunction of cough suppression mechanisms in the central nervous system^29^. The efficacy of neuromodulatory agents such as gabapentin^30^, pregabalin^31^, and P2X3 receptor antagonists (e.g. Gefapixant)^32,33^ has been reported. Further investigations are needed to determine whether administering these agents instead of central antitussives is more effective and has fewer side effects.

Regarding the occurrence of common adverse events, different trends were observed when the central antitussives were divided into opioid and non-opioid subgroups. Specifically, constipation was significantly and independently associated with the prescription of opioid antitussives, whereas nausea was independently associated with the prescription of non-opioid antitussives. These different trends may be influenced by the pharmacological effects of each central antitussive agent. For example, nausea is a well-known early adverse effect of opioid antitussives such as morphine, but it is also generally known that this symptom tends to diminish or disappear with long-term use due to the development of tolerance. This may explain why the odds ratio for nausea did not increase in patients receiving long-term prescriptions of opioid antitussives. Despite the mechanisms not being fully elucidated, our study is one of the first to demonstrate an association between the risk of common adverse events and the duration of central antitussive prescriptions in a large number of patients in a real-world setting. Therefore, it is essential to prioritize the treatment of the causative disease based on the anatomical diagnostic procedures recommended by the guidelines rather than relying on easily prescribed central antitussives. Despite concerns about adverse events, central antitussives remain an important option in selected clinical situations. When used properly, central antitussives can be effective treatments for cough, improving symptom control and quality of life. Clinicians should be educated on the prescription and monitoring of central antitussives.

This study has some limitations that should be considered when interpreting the findings. First, patients aged > 65 years were excluded because the database was derived from a health insurance association. To overcome these limitations, it is desirable to conduct future studies using databases containing diverse variables and comprehensive population coverage. Second, our analyses were based on prescription records using claims data, which may not reflect the actual use of central antitussives. However, since adverse events were linked to the prescription of central antitussives, we believe that the prescription frequency of central antitussives in this study reflects real-world clinical practice. Third, due to database limitations, only two major adverse effects could be assessed, while others such as drowsiness were not evaluated. However, the increasing proportion of reported adverse effects with longer treatment suggests that our findings reflect routine clinical practice to a considerable extent although prospective studies are warranted. In addition, this database does not include information on physicians’ specialty qualifications, which precluded an important investigation into prescribing intentions for central antitussives. Future studies using databases with more comprehensive information will be needed to address this issue. Finally, the relationship between cough severity and comorbidities was unclear. Therefore, we cannot interpret the reasons for long-term prescription of central antitussives. Newly collected data in routine clinical practice, rather than claims data, will further validate our findings.

In conclusion, this study revealed that central antitussives are frequently and persistently prescribed, with long-term use was linked to adverse events in patients with chronic cough. It is important to sufficiently evaluate treatable traits and provide systematic management, including consideration of the adverse event profiles of central antitussives, which may help promote their safer and more rational use for patients with chronic cough in routine clinical practice.

## Methods

### Study design and data source

This retrospective cohort study was conducted using data from the IQVIA Claims database (IQVIA-DB), a Japanese insurance claims database, in respect of which data IQVIA reserves all its rights^34^. It used a dataset of patients diagnosed with cough between August 2018 and July 2023 . The design and time windows used in this study are shown in Supplementary Figure S1a.

### Definition of subacute and chronic cough

The algorithm for defining subacute and chronic cough using claims data was modified based on previous studies^18,19,27^. First, we extracted records of ICD-10 codes or diagnoses for cough (Table 3) from August 1, 2019, to July 31, 2022. Since diagnostic information was recorded monthly, we included patients who had two or more cough diagnoses within three months after the index month (chronic cough) or at least one diagnosis within two consecutive months (subacute cough) (Supplementary Figure S1b).

**Table 3:** Definition of cough.

Type of cough	ICD-10 codes		Diagnosis name
Cough	R05 cough		
Whooping cough	A37 Whooping cough	and	Whooping cough due to Bordetella pertussis Whooping cough, unspecified
Psychogenic cough	F45.3 Somatoform autonomic dysfunction	and	Psychogenic cough
Cough variant asthma	J459 Asthma, unspecified	and	Cough variant asthma
Primary cough headache	G448 Other specified headache syndromes	and	Primary cough headache

ICD-10, International Classification of Diseases 10th revision.

### Definition of central antitussive

Drugs included as central antitussives and their definitions are listed in Supplementary Table S3. Discrimination between opioid and non-opioid antitussives was defined based on the combination of the “ATC code” (R05D1 or R05D2) and the “Ingredient name” (containing “codeine” or “dihydrocodeine”).

### Inclusion and exclusion criteria

**Fig. 3:**
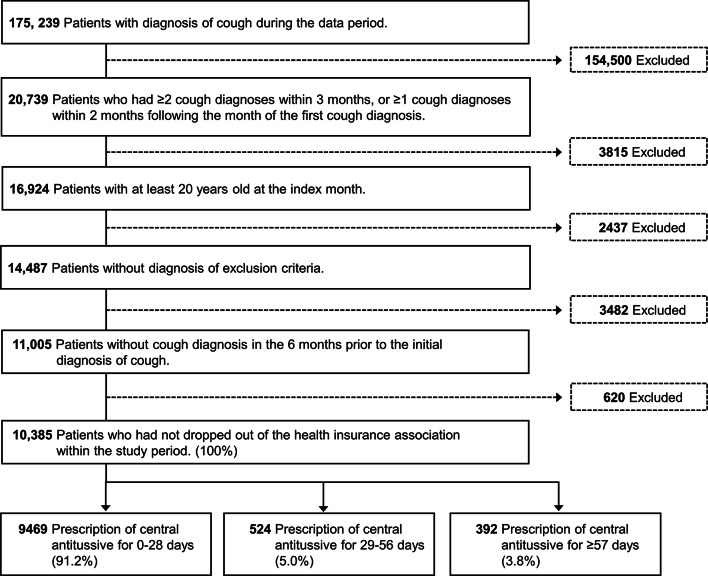
Flow of eligible patients.

The primary inclusion criteria for this study were patients with chronic cough aged ≥ 20 years who did not have a diagnosis of cough within 6 months prior to the index month and who did not drop out of the database during the study period (look-back and follow-up periods). Patients with underlying pathological factors, such as cancer or interstitial lung disease, or those taking angiotensin-converting enzyme inhibitors were excluded from the study. Those who met all the eligibility criteria shown in Fig. 3 were included in the analysis. All the inclusion and exclusion criteria are presented in Supplementary Table S4.

### Outcomes and variables

The primary outcome was the number of days that central antitussives were prescribed during the follow-up period. The secondary outcomes were the frequency of constipation (defined by ICD-10 K59.0) and nausea (defined by ICD-10 R11.0), which are common adverse events associated with central antitussives.

The variables summarised in this study were patient age, sex, department, facility size in the index month, medical history/comorbidities, and treatment information during the observation period. The medical history and comorbidities included in the study were physician diagnosed BA/CVA, chronic obstructive pulmonary disease (COPD), chronic bronchitis, AR/CRS, VCD, OSA, and GERD obtained from IQVIA claims database. Furthermore, Medications such as bronchodilators, oral corticosteroids (OCS), inhaled corticosteroids (ICS), ICS/long-acting β2-agonist (LABA), ICS/LABA/long-acting anticholinergics (LAMA), leukotriene receptor antagonists (LTRA), biological agents, proton pump inhibitors (PPIs), functional gastrointestinal agents, histamine H1 receptor antagonists, and 14-/15-membered ring macrolides were included. Detailed definitions of the diagnoses and treatments are provided in Supplementary Table S5.

### Statistical analyses

To demonstrate the distribution of patient characteristics, the mean (SD) or median (interquartile range [IQR]) was calculated as appropriate for continuous variables, and the frequency and percentage (%) were calculated as summary statistics for categorical variables. Chi-square tests and analysis of variance (ANOVA) were performed to determine the differences in characteristics between groups.

Descriptive statistics for the primary outcome included the number of days of central antitussive prescription in weekly units during the follow-up period. The number of days prescribed for central antitussives was categorized as 0–28, 29–56, and ≥ 57 days, and the co-occurrence patterns of comorbidities and medications in each category were tabulated. We included three diagnoses that are reportedly closely associated with cough: BA/CVA, AR/CRS, and GERD. Other diagnoses, such as OSA, have also been reported to be associated with cough^10^; however, we excluded this from the pattern analysis because of the small number of cases and the complexity of the pattern, which makes it difficult to interpret. Statistical hypothesis testing was not performed to analyse the primary outcome. For the secondary outcome analysis, an ordinal logistic regression model was used to explore the differences in the number of comorbidities and medications (none, one, two, and three) according to the number of prescribed days of central antitussives. To examine the association between the presence of constipation, nausea, and the number of days central antitussives were prescribed, the data were classified into three categories, and multivariable logistic regression analysis was conducted. In the logistic regression analysis, the outcomes that occurred during the follow-up period were entered as the dependent variables, and the category of days of central antitussive prescription was the independent variable. Covariates included the history of outcomes that occurred during the look-back period (constipation/nausea), age, sex, total number of months of cough diagnosis, and comorbidities (BA/CVA, AR/CRS, and GERD). Adjusted ORs, their 95%CIs, and *P* values were also calculated. For sensitivity analysis, we also analyzed data when the number of days of central antitussive prescriptions was classified into four categories (none, 1–28, 29–56, and ≥ 57). Additionally, the Hosmer–Lemeshow test was used to evaluate the goodness of fit of the model. The two-sided significance level for all statistical tests was set at 5%, with a 95% CI. In the subgroup analysis, we conducted secondary outcome analyses of central antitussives by subgrouping them into opioid and non-opioid groups. Statistical analyses were performed using R version 4.4.0 (R Foundation for Statistical Computing, Vienna, Austria). The results were reported according to the recommendations of “Reporting of studies Conducted using Observational Routinely collected Data” (RECORD)^35^.

## Supplementary Information

Below is the link to the electronic supplementary material.

Supplementary Material 1

Supplementary Material 2

Supplementary Material 3

Supplementary Material 4

Supplementary Material 5

Supplementary Material 6

Supplementary Material 7

## Data Availability

The data that support the findings of this study are available from IQVIA G.K. but restrictions apply to the availability of these data, which were used under license for the current study, and so are not publicly available. Data are however available from the corresponding authors upon reasonable request and with permission of IQVIA.
