# Supplementary figures and images for: Real-world survey on utilization of central antitussives and its health impact in patients with subacute and chronic cough in Japan
Source: Sci Rep. 2025 Dec 8;16:1145. doi: 10.1038/s41598-025-30832-6 (PMC12789445; doi:10.1038/s41598-025-30832-6)

**Supplementary Table S5.** Definition of treatment for each comorbidity


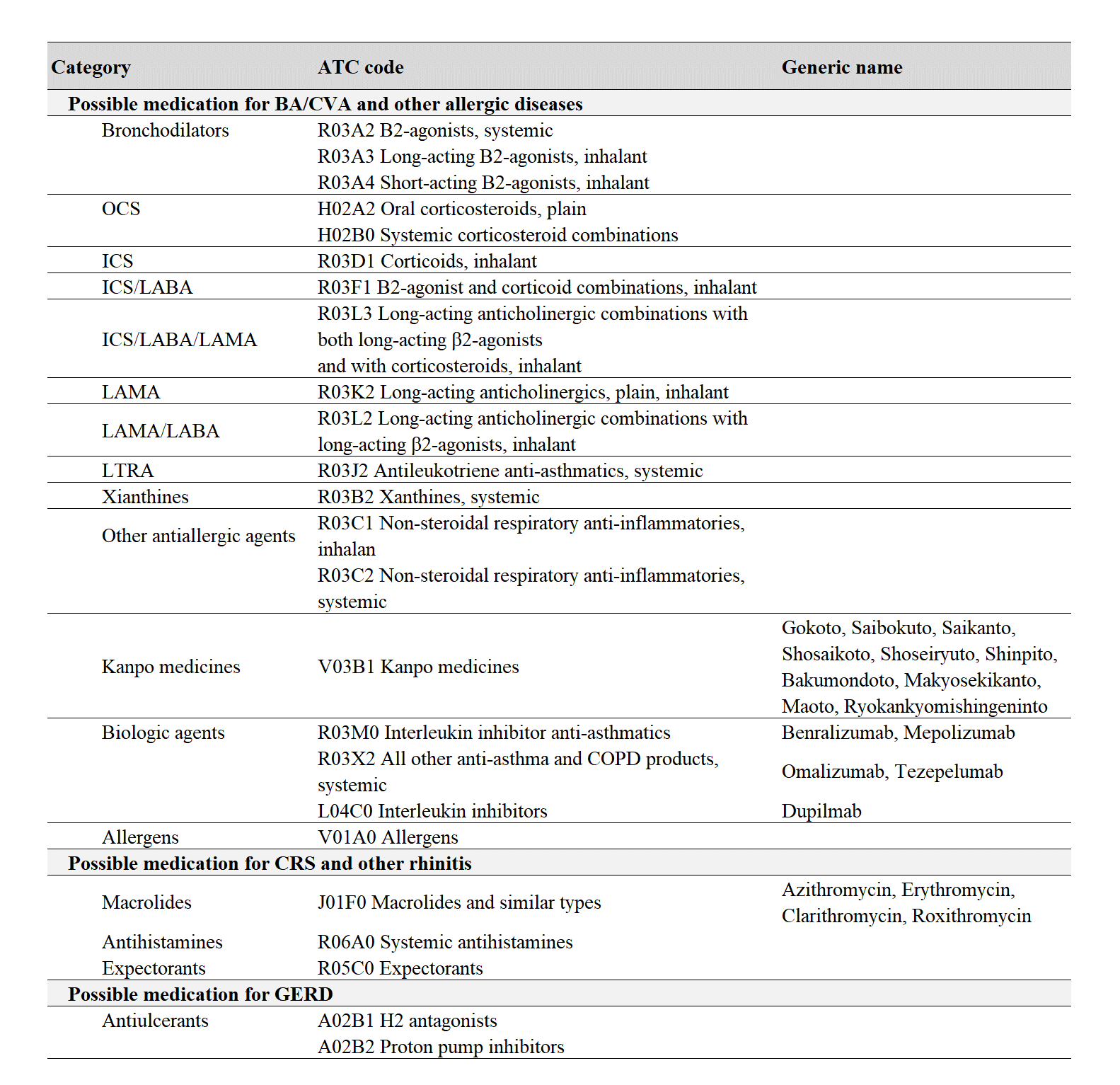

Supplement: Supplementary file 1 — Supplementary Material 1 [file 41598_2025_30832_MOESM1_ESM.docx]

**Supplementary Table S4.** Inclusion and exclusion criteria


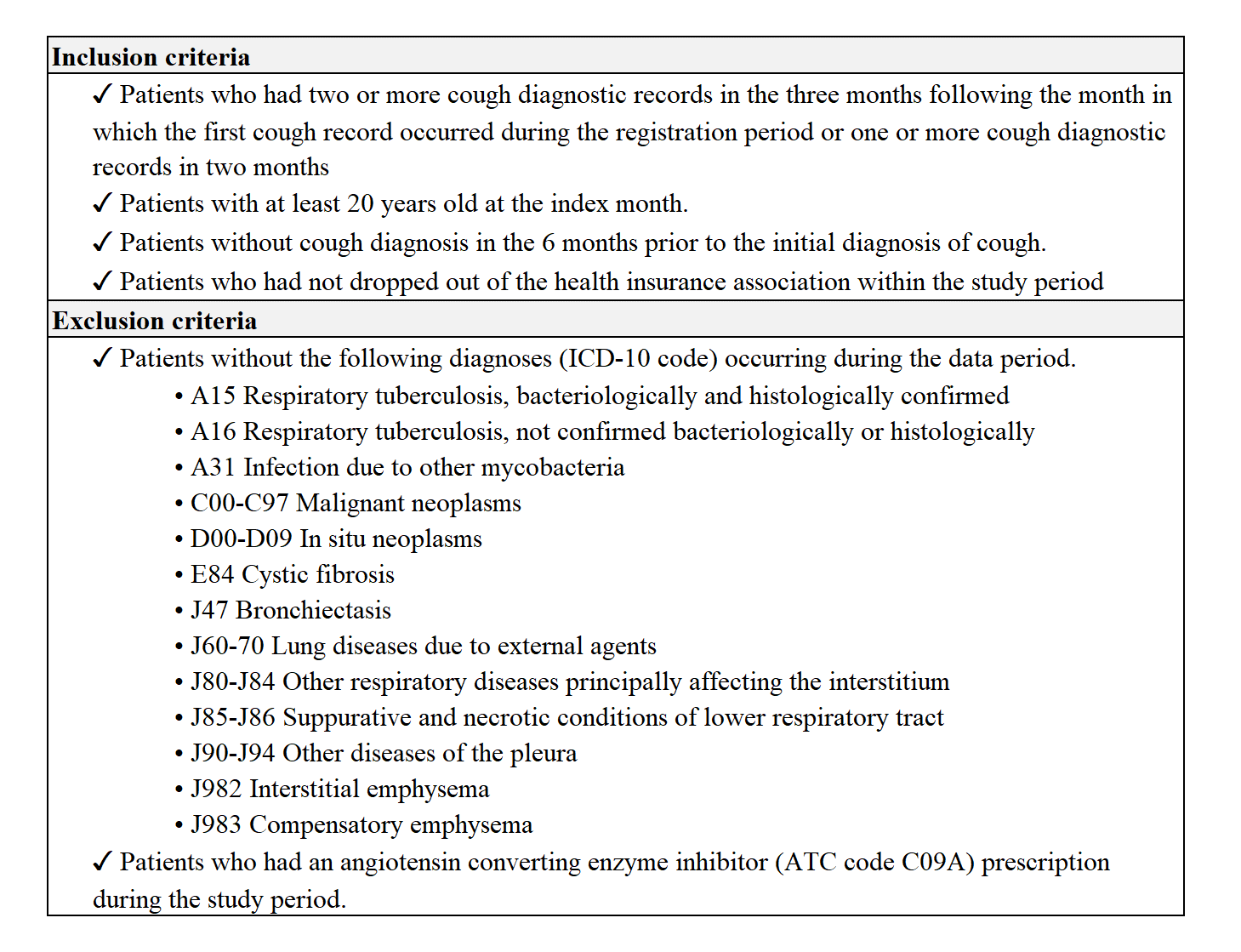

Supplement: Supplementary file 3 — Supplementary Material 3 [file 41598_2025_30832_MOESM3_ESM.docx]

**Supplementary Table S3.** Definition of opioid and non-opioid central antitussive


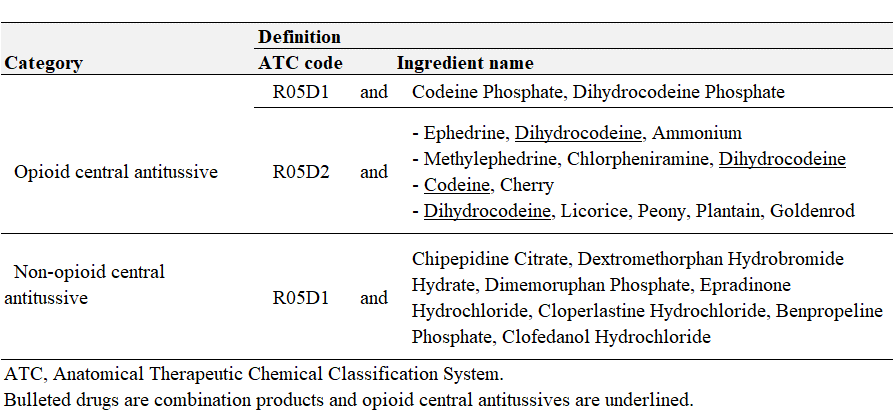

Supplement: Supplementary file 6 — Supplementary Material 6 [file 41598_2025_30832_MOESM6_ESM.docx]
