# Supplementary material for: Real-world survey on utilization of central antitussives and its health impact in patients with subacute and chronic cough in Japan
Source: Sci Rep. 2025 Dec 8;16:1145. doi: 10.1038/s41598-025-30832-6 (PMC12789445; doi:10.1038/s41598-025-30832-6)
Supplement: Supplementary file 2 — Supplementary Material 2 [file 41598_2025_30832_MOESM2_ESM.docx]

**Supplementary Table S1.** Descriptive statistics for other comorbidities


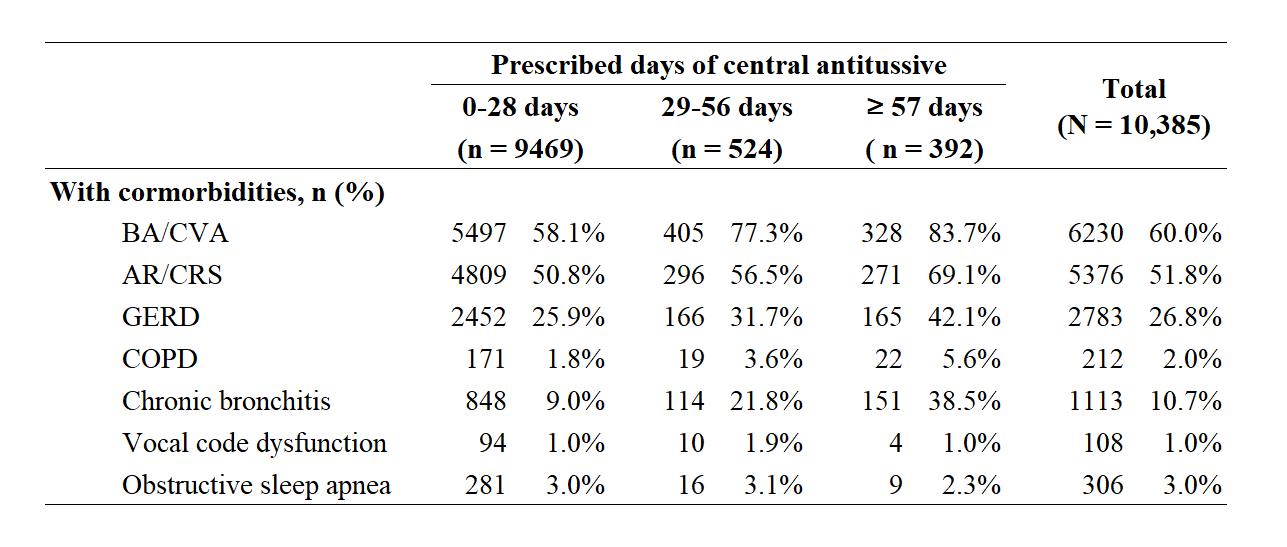


AR, allergic rhinitis; BA, bronchial asthma; CRS, chronic rhinosinusitis; CVA, cough variant asthma; GERD, gastroesophageal reflux disease
