# Supplementary material for: Real-world survey on utilization of central antitussives and its health impact in patients with subacute and chronic cough in Japan
Source: Sci Rep. 2025 Dec 8;16:1145. doi: 10.1038/s41598-025-30832-6 (PMC12789445; doi:10.1038/s41598-025-30832-6)

**Supplementary Figure S1.** Study design and time window

a. Black bars represent the entire data period and brown bars represent the enrollment period. One year after the Index month was defined as the follow-up period, and the previous half year of the Index month was defined as the look-back period. b. Index month was defined as the month in which the first diagnosis of chronic cough was recorded during the enrollment period. In addition, the index month was defined as the month in which the second cough diagnosis was recorded when there were two or more cough diagnoses within 3 months following the month in which the first cough diagnosis was recorded.


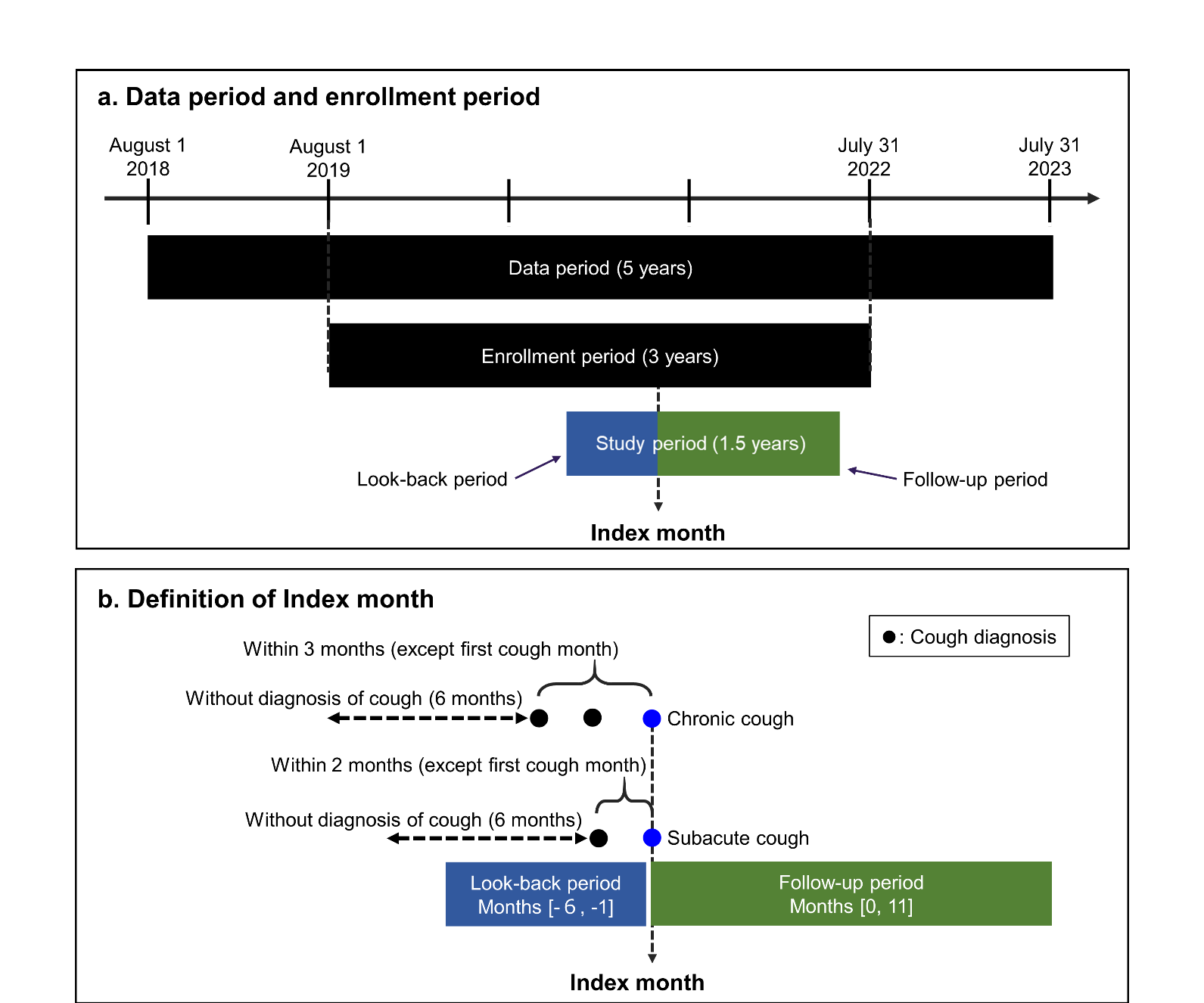

Supplement: Supplementary file 4 — Supplementary Material 4 [file 41598_2025_30832_MOESM4_ESM.docx]
