# Supplementary material for: Real-world survey on utilization of central antitussives and its health impact in patients with subacute and chronic cough in Japan
Source: Sci Rep. 2025 Dec 8;16:1145. doi: 10.1038/s41598-025-30832-6 (PMC12789445; doi:10.1038/s41598-025-30832-6)

**Supplementary Table S6.** Associations between prescribed days of central antitussives and adverse events from multivariable Logistic regression analyses (sensitivity analysis)


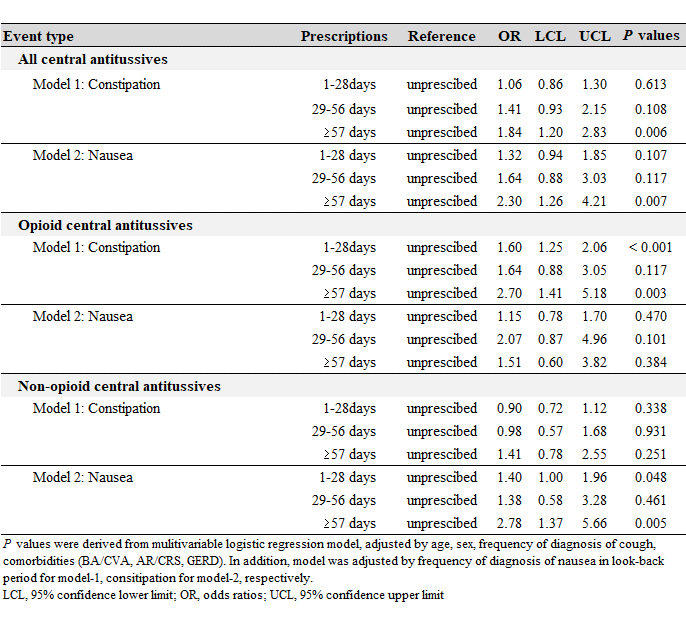

Supplement: Supplementary file 7 — Supplementary Material 7 [file 41598_2025_30832_MOESM7_ESM.docx]
